# A Qualitative Analysis of the Perceptions of Stakeholders Involved in Vector Control and Vector-Borne Disease Research and Surveillance in Orinoquia, Colombia

**DOI:** 10.3390/tropicalmed9020043

**Published:** 2024-02-06

**Authors:** Gloria Isabel Jaramillo-Ramirez, Maria Claudelle Tacugue, Grace M. Power, Rimsha Qureshi, Frederik Seelig, Juliana Quintero, James G. Logan, Robert T. Jones

**Affiliations:** 1 School of Medicine, Universidad Cooperativa de Colombia, Villavicencio 500017, Colombia; 2 Department of Disease Control, London School of Hygiene & Tropical Medicine, Keppel Street, London WC1E 7HT, UK; claudelle.tacugue@nhs.net (M.C.T.); grace.power@bristol.ac.uk (G.M.P.); rimsha.qureshi@ukhsa.gov.uk (R.Q.); frederik.seelig@lshtm.ac.uk (F.S.); juliana.quintero@lshtm.ac.uk (J.Q.); james.logan@lshtm.ac.uk (J.G.L.); robert.jones@lshtm.ac.uk (R.T.J.); 3 MRC Integrative Epidemiology Unit, Population Health Sciences, Bristol Medical School, University of Bristol, Bristol BS8 1QU, UK; 4 Global Vector Hub, London School of Hygiene & Tropical Medicine, Keppel Street, London WC1E 7HT, UK; 5 Division of Population Health and Internal Medicine, Fundación Santa Fe de Bogotá, Bogotá 110011, Colombia

**Keywords:** public health, vector-borne diseases, vector control

## Abstract

Colombia has a tropical climate and environmental conditions that favour the circulation of most of the known vector-borne diseases (VBDs). Protocols have been established and implemented to address the threats of these diseases, but they are for country-wide use and do not take into consideration the nuances of the different environments of the country. Almost the entire population is vulnerable to infection with one or more VBD. This study aims to characterise the perceptions and experiences of stakeholders involved in vector control and VBDs in the Orinoquia region in Colombia. Two panel discussions, and 12 semi-structured interviews, were conducted. Experts from the Colombian National Health Institute (INS), health secretaries from Meta, Guaviare and Vichada Departments, academic researchers, and individuals from private vector control companies participated. All sessions were recorded, transcribed, and translated, and then subject to thematic analysis. Three major themes emerged: involvement, limitations, and recommendations. Results showed that participants are engaged in vector surveillance activities, education, and vector control research. Participants focused on problems of disjointed efforts towards VBD control between health secretaries and the health ministry, as well as societal issues, such as socioeconomic, cultural, and political issues, which became the rationale for the lack of vector control resources. Responses in the panel discussions and interviews overlapped in opinions, and suggested that vector control could be improved through better communication between vector control bodies, strengthened engagement with vulnerable communities, more collaborative actions, and a more balanced distribution of resources.

## 1. Introduction

Colombia is a tropical country with a variety of climatic and environmental conditions that support the survival of arthropod vectors of disease. Malaria is still reported in the Pacific and Amazon regions [1], and American trypanosomiasis is also endemic in almost the whole country [2,3]. Two-thirds of the Colombian territory has favorable geographical and climatic characteristics for the reproduction of the vector and the presence of the disease [4]. According to the geographical distribution of acute Chagas in 2022, historically known risk scenarios are maintained in part of the Colombian Orinoquia in departments such as Casanare and Arauca, part of the Andean region in the departments of Santander and Norte de Santander, and a new risk scenario was recently identified in the Caribbean region in the departments of Cesar and Sucre. Confirmed chronic cases were reported in high proportion in departments such as Casanare, Cesar, Santander, Norte de Santander y Arauca [5]. In addition, leishmaniasis is found in a large part of the country, particularly in sylvatic foci [3,6]. Among the Aedes-borne diseases, Colombia is one of the countries in the Americas most affected by epidemics of dengue, with over 127,500 cases being reported in an epidemic in 2019 and one of the highest case fatality rates in the region [7,8]. The first laboratory-confirmed autochthonous cases of chikungunya were reported in September 2014 [9], and less than a year later the Zika virus arrived [10]. By the end of the Zika outbreak in early 2018, over 98,800 suspected cases of infection had been reported by the Pan American Health Association (PAHO) [11]. The co-circulation of arboviruses poses a continued threat to the population, and creates a challenge for case confirmation because of similarities in the clinical presentation of arboviral diseases. 

Colombia has many organisations involved in the control of arthropod vectors, including both public and private universities, and international health agencies. These groups help the Colombian government in vector control and vector-borne disease control. Moreover, relevant research groups such as Programa de Estudio y Control de Enfermedades Tropicales (PECET) [12], Centro Internacional de Entrenamiento e Investigaciones Medicas (CIDEIM) [13], and Centro de Investigación Científica CAUCASECO (CIC) [14], are dedicated to the research of transmissible diseases. International agencies such as the CDC or World Health Organisation (WHO) are involved too.

**Figure 1:**
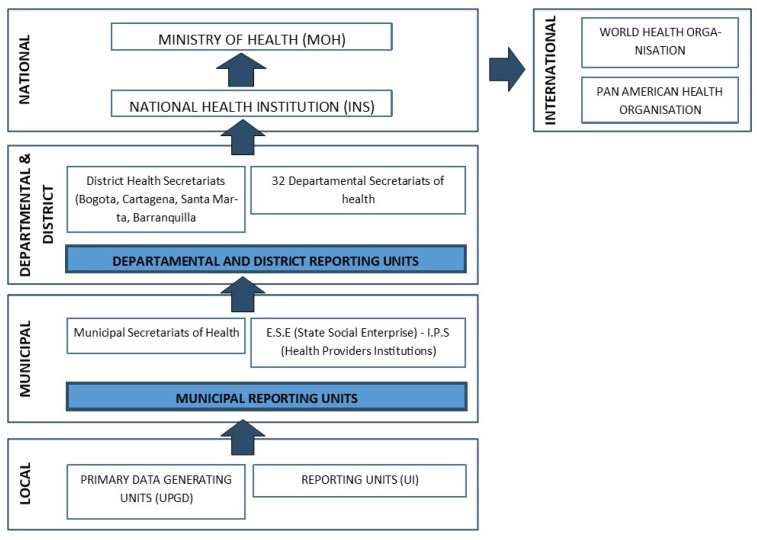
The public health surveillance system in Colombia. Adapted from National Health Institute protocols [27].

The principal administrative agency overseeing national policies for public health surveillance and vector control in Colombia are the Ministry of Health and Social Protection (MHSP) and the National Health Institute (Instituto Nacional de Salud—INS). The INS is responsible for overall public health surveillance, as well as the monitoring of disease vectors. It coordinates and issues guidelines to the Public Health Surveillance System (SIVIGILA), which provides timely information on events that are of interest to public health [15] (Figure 1). The INS also issues protocols for disease control and management of intervention programmes for multiple diseases. These protocols include disease information and country statistics, surveillance measures and objectives, case definitions, and analysis of data. In addition, the protocols include an outline of the roles of personnel and the actions to be taken by each specific group, such as laboratory procedures for identifying the serotypes in circulation [16].

Vector control activities include the distribution of insecticide-treated bed nets to control malaria in endemic areas, as well as insecticide spraying and control of larval sites [17,18,19]. Bed net distribution and indoor residual spraying with pyrethroids are also part of the National Leishmaniasis Program, and spraying campaigns are used against triatomine vectors for Chagas disease control [20]. Control of *Aedes* mosquitoes is based on the use of chemical and biological insecticides in domestic water containers, and ultra-low volume spraying in public spaces [21]. The World Mosquito Program has a presence in Colombia and is using self-sustaining *Wolbachia* to introduce naturally occurring *Wolbachia* bacteria to *Aedes* mosquito populations to help combat arbovirus transmission. Following initial small-scale releases, large-scale trials are now underway in Bello and Medellin, and three further cities in western Colombia have joined the initiative [22].

Despite these efforts, vector-borne diseases (VBDs) remain a threat, and more than half of Colombia’s population is at risk of infection [2,3,6,8]. Like in other endemic countries, economic and socio-political issues, and the state capacity to address them influence the transmission of VBDs [9]. State capacity has been characterized by Homer-Dixon to consist of eight factors, distributed in two groups: (i) internal state attributes, such as human capital, instrumental rationality, coherence, and resilience; and (ii) state interactions with society, such as autonomy, fiscal resources, reach and responsiveness, and legitimacy. All these factors influence the distribution and determinants of health-related states [23,24]. In Venezuela for example, the re-emergence of some VBDs has been attributed to political destabilization and economic collapse. Studies have revealed that the rise of these diseases in Venezuela is due to shortages on insecticides and medicines, food insecurity that leads to malnutrition, human migration, poor support to government health workers, and reduction of epidemiological surveillance and reporting activities [25]. In Colombia, there are other social problems that may affect the rise of VBDs: governmental instability in some places diminishes the confidence and commitment to collective welfare and is reflected in the participation of the community in health and vector control programmes [26].

Unfortunately, in many endemic areas of Colombia, community participation in vector control programmes is low, so infestation rates and the risk of epidemics remain high. This work aims to provide a broad picture of the challenges faced in vector control, allowing not only an understanding of the root of the problem but also the establishment of starting points necessary for improvement. In this study, we held panel discussions and interviews with stakeholders from different institutions and from different departments of the Orinoquia region of Colombia (Arauca, Casanare, Meta, Guaviare, and Vichada), with the purpose of characterising the experiences of vector control of these stakeholders and to record any limitations that they perceive.

## 2. Methods

### 2.1. Study Site

**Figure 2:**
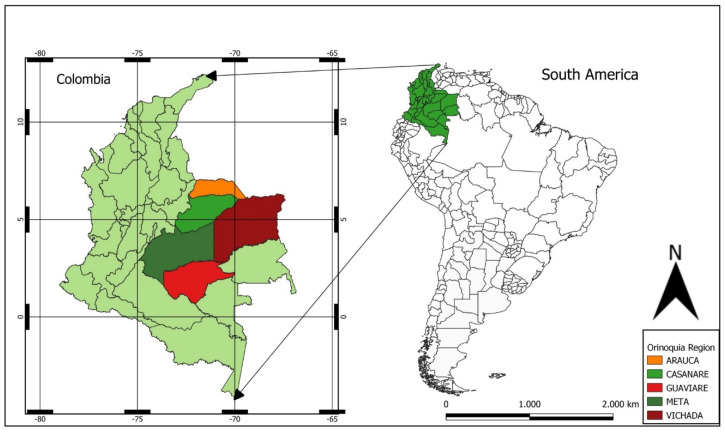
Location in Colombia of interviews and panel discussion participants. Map made by the authors in QGIS 3.26.3 Cloud free software (https://www.qgis.org/es/site/ (accessed on 6 July 2023)).

The study was carried out in the province of Orinoquia Colombiana (Colombian Orinoquia), located in the east of Colombia (Figure 2). This is a region characterized by plains with large areas for livestock and is one of the most important zones for petroleum extraction of Colombia. Average temperatures range from 22–35 °C throughout the year, with almost nine months of rainy season, which creates good breeding conditions for the vectors of arboviruses. The panel discussions were conducted in the first regional and international meeting of experts in control of arboviruses transmitted by *Aedes aegypti*, organized by Universidad Cooperativa de Colombia (Villavicencio branch). Members of the public were invited to join the guest panel made up of health workers, experts in vector control and health secretary workers of the region, as well as international researchers. In addition to this session, some of the panellists were interviewed individually.

### 2.2. Study Design

A qualitative study was carried out to understand the perceptions of the personnel in charge of vector control in the eastern part of the country and of those who carry out research on the subject. We used interviews and panel discussions, which are among the most commonly used methods of data collection employed in qualitative research and allow for a deeper understanding than would be obtained from more quantitative methods, such as questionnaires [28]. To obtain detailed insights from individual participants, we designed interview questions that were open-ended, neutral, and understandable, and which were likely to yield as much information as possible. Similar questions were used for the panel discussion sessions, which further allowed for group dynamics to generate qualitative data and encourage discourse between participants [29]. Both in the interviews and in the panel discussions, the participants had the opportunity to freely interact and express their opinions [30].

For interpretation of the data collected in interviews, we used thematic analysis. This is a systematic process of coding information, examining its meaning, and providing a description through the creation of themes. In particular, we aimed to identify common themes that would allow visualization of the main problems in Colombian Orinoquia and allow for viable recommendations to be suggested [31,32,33].

### 2.3. Data Collection

For the collection of information, semi-structured interviews and panel discussions were used. For semi-structured interviews an interview guide was followed (Additional File S1) that covered five sections for all interviews: (i) basic information about the interviewee, (ii) characteristics of vector control and surveillance tools, (iii) participation of the different actors, (iv) limitations/barriers of vector control actions, and (v) knowledge gaps. The guiding questions provided some structure to in-depth discussions. Twelve individuals with knowledge of vector control and working in different roles were interviewed (Table 1). Some of these experts also participated as panellists in the event described below. The interviews mostly lasted under an hour and were held in Spanish or English. They were held in private and were conducted between one or two researchers plus the interviewee. All sessions were audio-recorded using a digital device, and notes were taken.

Panel discussion groups took place within the first regional and international meeting of experts in control of arboviruses transmitted by *Aedes aegypti*, organized by Universidad Cooperativa de Colombia (Villavicencio branch), held on 18 September 2019 (Additional File S2). The event was divided into three panel discussions: (1) the governmental entity, the private sector, and higher education against arboviruses and their control; (2) health service providers in the clinical management of patients with arboviruses transmitted by *Aedes*, and (3) the role of society in the control and prevention of arboviruses transmitted by *Aedes*. For the analysis of this article, only panels (1) and (3) were considered.

The panel discussion sessions were held in Spanish and were of approximately 1 h and 20 min duration. They consisted of invited participants and an informed moderator. Each panel had some guiding questions that allowed the experts to interact, and the audience was also allowed to intervene. The latter included professionals from the health secretariats of the Orinoquía departments, staff from clinics and hospitals in the region, and students from the medical and nursing schools. Individuals from affected communities were not present but the participating anthropologists were able to share insights form working with these communities. The full list of guideline questions used in the sessions is provided as a Supplementary File (Additional File S3). Note that only a subset of these questions was asked in each session due to some questions not being of relevance to all participants, and further questions were used to encourage elaboration.

### 2.4. Sample Characteristics

Participants were purposely sampled based on their background in vector control in Colombia and their interest in sharing their opinions. Twenty participants were invited to take part in the interview sessions. To maximise efforts in getting all participants to engage, a reminder email was sent out to encourage involvement two weeks after the initial email had been sent. Twelve respondents accepted the invitation and the remainder refused or did not respond. The participating individuals had varying career backgrounds, both from the public and private sectors, which provided a range of perspectives and insight during the sessions (Table 1). The characteristics of participating expert panellists are shown in Table 2.

### 2.5. Data Analysis

All recorded panel discussions and interviews were fully transcribed, and the Spanish text was translated into English. The English transcriptions were then coded in NVivo 12 (QSR International) for content analysis, which explores text and is used to build structure to collected data [34]. By drawing important information from the textual data, and structuring it, content analysis creates new knowledge [35]. In this case, the aim was to provide a structure by classifying interview responses into specific categories. A deductive content analysis approach was used [36,37]: pre-defined categories and sub-categories were established based on the nature of the questions asked to participants and the research questions being investigated, but emerging categories were also identified once the researchers had become familiar with the transcribed text, and these were used when the text was analysed (Table 3). The initial analysis was carried out by MCT and RQ, who led data collection and complemented the results with field notes. These researchers broke down the text into meaningful units, labelled with codes. They then checked whether all important aspects of the content had been captured by the codes [38]. The coded text was then sorted into categories. For rigor, the analyses were checked by GIJ and JQ [36,37]. Finally, discussions on the findings were held with all authors for the development of the manuscript.

### 2.6. Ethical Approval

The London School of Hygiene & Tropical Medicine Research Ethics Committee approved the investigation; reference numbers: 17,456, 17,720, and 21,964. Each participant provided written informed consent to take part.

## 3. Results

### 3.1. Involvement in Vector Control

Participants described the overall process of vector control as following a systematic route that begins with a close observation of the locations where a VBD case has been detected. Cases are usually identified by members of the public surveillance group and laboratories in the Departmental Health Secretaries. Specific control procedures are then carried out by programmes under the ‘Vector-borne disease VBD’ group (Enfermedades transmitidas por vectores ETV in Spanish), which participants described as involving principally chemical control methods. Participants from the department of Guaviare and Meta described the division of activities during the onset of an outbreak:

*“The Entomology group (that is part of the Departmental Health Secretaries) does the characterization. Let’s say we call it pre-intervention actions: characterize the vector, propose a control option and then the vector programme implements the actions and we also evaluate the impact of those actions.”*

—I2

*“Control is done by the VBD group; they manage different tools such as education of the community, sensitizing the community, sensitizing health personnel…”*

—I5

The participants highlighted that surveillance of the vector is crucial to prioritising areas of work by vector control programmes.

*“Our activity is based on detecting the trends of increase or decrease in order to be able to work in coordination with the prevention programme. Once the tendency to increase is detected, the manager of the promotion and prevention office is notified and he, with the vector control team, designs the intervention strategies.”*

—I1

Participants noted the particular focus on those areas that are difficult to reach. For example, the department of Guaviare in south-central Colombia has many remote and rural areas with indigenous communities, and it is important that actions are taken to reach those localities where VBD cases are being detected. For the case of Guaviare, a field trip is usually planned in order to obtain the information needed before any form of control is implemented. The planning process itself can take up to two weeks, and the response after that is typically conducted with limited resources. These resources could be money, availability of transport, and the materials needed for biological testing in the field and laboratory.

*“Sometimes, when the areas are very far away, what we do is take all the available supplies for control, this is ideal, and this is what happens many times. Other times, because there are no resources, we depend a lot on what the vector office at the Health Secretary gives us.”*

—I2

In addition to describing these activities and the sequence of events that occurs following case detection, most of the participants also expressed their involvement in vector control in terms of their research and other contributions. The research activities made by health secretaries are important to improve vector control programmes, and most of their studies are funded by the ministry of health and the national health institute. For example, a representative from Guaviare’s Health Secretary laboratory revealed that they have conducted trials with essential oils extracted from *Cynodon nlemfuensis*, also known as star grass. The essential oils extracted from this plant, which is an introduced species that grows widely in Colombia, can be taken to incorporate into repellents. An interviewee from the private sector from Bogotá shared their successes in the development of vector control technologies, including domestically-produced mosquito nets and awnings. They had also conducted research into wearable repellents and the impregnation of permethrin into clothing, in particular uniforms for the military.

*“We use these technologies with the Colombian army, in order to protect the soldiers in areas where leishmaniasis and malaria occur.”*

—I6

The responses also revealed that participants held contributions towards the education of students as being of great importance.

*“…We need more resources and more research. We are working on it… I think that the vector control programmes haven’t failed, but the problem is the scale. At the university we are working to find common solutions to improve these control programmes…… We are working with the public and private sectors, not only in vector control but also in the correct diagnosis of patients.”*

—PD1

### 3.2. Limitations of Vector Control in Colombia

Participants in the interviews and panel sessions discussed the obstacles they had faced and provided their views on the country’s shortcomings in vector control activities.

#### 3.2.1. Delayed Responses to Vector Outbreaks

Several participants indicated that there were decisions made higher up in the vector control hierarchy that were inadequate and limited their ability to respond to outbreaks. A particular problem expressed was the tendency to react to emergencies rather than to plan ahead and to devote sufficient time to structural problems. This emerged as a recurrent theme in both the panel discussions and the interviews.

*“We should already be doing actions prior to the outbreak, which unfortunately we spend as firefighters only putting out fires.”*

—I5

Whether before an official alert is issued, or as soon as a case is detected, participants were concerned about tackling outbreaks in a prompt manner.

*“(In some cases) we are seeing the reflection of the first cases in the November-December SIVIGILA and we are responding at the end of January, (then) intervening in March and April, so the opportunity for a response I would say is appalling.”*

—I2

*“We know that the outbreak begins in the months of March or April because we are not already working to mitigate that outbreak. Ggenerally what we are doing is waiting for the outbreak to appear before we start intervening.”*

—PD1

#### 3.2.2. Lack of Resources

Study participants indicated that there are limitations in staff and other resources in Colombia for both vector control and research on VBDs. One participant described entomological surveillance and diagnosis to be a costly task that requires baseline technology and continuous investment into research to maintain diagnostic gold standards. As the research and vector control community are given what they considered to be minimum resources, priority was largely given to vector control tools at the expense of research.

Some specifically noted a poor distribution of resources, which limited both the training available to teams working to prevent VBDs and the funds available for research and surveillance purposes. For example, some participants indicated that resources and experts are concentrated in Bogota, despite VBDs being more prevalent in rural settings. This disproportionate distribution limits the capacity of the teams in those more remote places.

Other issues that participants perceived as limiting vector control were corruption, inappropriate use of funds, and political instability. Colombia has what are known as ‘regalías’, or royalties, in each department. These royalties are the payments made by oil and mining companies to the Colombian State for exploiting deposits of non-renewable natural resources. For example, oil companies deliver to the state between 8% and 25% of the value of crude oil production. These funds are destined to solve the unsatisfied basic needs of the departments and municipalities, such as basic education, health, and drinking water, as well as to finance large projects that bring development to the region [21]. One participant perceived that these funds should be made available for research and vector control activities, but were instead used on more visible matters:

*“They prefer to give this money to do construction… (which indicates) that the governor or that the political leadership is doing something.”*

—I2

*“The truth is, there are no resources assigned for research, despite the fact that there is money (and a need) to conduct research.”*

—I1

#### 3.2.3. Administrative Discontinuity

The issue of political instability has a particular consequence in Colombia as it is accompanied by changes in personnel in health secretaries. Across all departments represented in this study, the subject of staff continuity within the administration of vector control programmes was discussed. According to one of the participants, the current programme set by the Ministry of Health is a yearly information and policy update on a national scale, alongside which technical assistance and training is provided at a local level. However, if personnel are replaced with each change of government, and trained workers are not being retained, it is necessary to hire and train new staff each year.

*“So, we need to make sure that each local state structure has a minimum of one person responsible for the training, and we need that this person being a public servant, not on the call of the government in power. Many of them have been working for many years, and there are reaching nearly the point of retirement. So, we need a least one person in each town, who is trained yearly.”*

—I11

Attention was also brought to the inefficient hiring processes and the issue of fixed-term contracts. It was clear that absence of staff at critical times and the need for repetitive training of new employees impacts the efficiency of the implementation of vector control activities across the country. Participants from three different departments spoke on this matter.

*“A staff member is on contract for six months, then a different employee comes. You have to adapt and get to know them, which does not generate any long-term engagements that allow you to implement organisational strategies.”*

—PD1

*“The entomologists are very poorly linked; there are very few in administrative careers and a few in provisional staff, and there are many others that are on contracts. These contracts (may be) of three or six months, so many times when an outbreak appears there is no one to attend.”*

—PD1

*“I teach someone to spray in the walls (with insectide to protect) against malaria during one year. The next year this guy is changed and you have to train again the new person that is coming into the vector control programme. And this is very difficult…”*

—I6

#### 3.2.4. Communication between Institutions

Related to the theme of discontinuity within an institution, some participants described a lack of cohesion between different bodies, such as between governmental bodies, medical professionals, and communities. For example, one participant pointed out misalignment between the guidelines provided to them and the decisions made by the VBD group. The same issue is seen in the provision of chemical control, where those who work in laboratories provided insecticide resistance reports to the INS, only for these to be apparently disregarded. Another participant reported a lack of evaluation of interventions and poor use of information within the vector control community:

*“The INS provides the information for action; the MHSP sometimes only operates with general guidelines but does not conduct follow-up actions or does not survey the (impacts of the) actions implemented”*

—I12

As a consequence of the lack of responsiveness to information, the most effective and up-to-date strategy is not being used to tackle the issue of VBDs. Participants believed that strengthening the capacity of programme management bodies at both national and subnational levels would be essential for the success of vector control.

Another view expressed in the interviews was that vector control was still very much concentrated on the public health sector alone. Whilst there was a recognition of a strong collaborative relationship between the environmental sector and the INS, a participant indicated that further collaborative work would be beneficial, and that there was a need for the housing and public services sectors to work together to reinforce vector control activities. The lack of inter-sectoral engagement seemed of particular concern in rural settings, where vector control is exacerbated by the lack of resources and a delayed consolidation of VBD data.

#### 3.2.5. Community Participation

A further concern discussed in the interviews and panel sessions was communication with inhabitants. Participants perceived a sense of normality created around VBD outbreaks in the community due to their frequency and being prevalent for years. Therefore, communities within Colombia do not take sufficient preventative measures and rely heavily on the government to implement policies and vector control programmes. This has generated an important need for risk communication to take place.

*“… specifically for diseases that are urbanized, the people see these events as very common diseases. This is reflected in the environmental control activities carried out. The people think that these diseases are inevitable.”*

—I12

A further challenge to effective community participation is the diversity of the communities present in Colombia. The country has many indigenous communities, almost 80% of which live in rural areas and have their own languages and practices [22], particularly medicinal practices in the form of herbal remedies. As a result, engagement with these communities has been a challenge for vector control organisations and there is a need to better understand the characteristics of each of the communities in order to implement suitable vector control strategies. Ineffective communication with these communities creates a barrier that hinders community participation in the prevention of VBDs.

*“The attitude of the indigenous person towards care at the time they have their disease does not allow the outbreak to be controlled… The indigenous people (prefer to manage disease) with their natural medicine, and the cases are increasing every time.”*

—I5

*“Almost all the ethnic groups that we have already have a way of practicing their own medicine, and the strategies that we use to bring them better health and prevention are not very well received.”*

—PD1

### 3.3. Participant Recommendations

Participants provided their own recommendations for the improvement of vector control and responses to VBD outbreaks. Some of these recommendations arose in responses to questions asked by the public in the panel discussions.

### 3.4. Sustainable Actions within the Communities

Some participants expressed the need for community-wide behavioural change, taking into account all cultures and practices, without overstepping boundaries. One suggestion was to implement the World Health Organization (WHO) Communication for Behavioural Impact (COMBI) strategy, which serves as a toolkit in an outbreak response. It is a planning framework that uses communication strategically to achieve positive, long-term behavioural and social results that eventually become incorporated into daily routine [23].

*“(To involve) indigenous communities in the strategies, it must start from within them; sometimes one has to impose actions of Western culture and this does not take into account their own particularities.”*

—PD1

*“(We have to) understand people too. Not even many people, all people: mestizos, indigenas, afrocolombianos, all.”*

—I7

### 3.5. Strengthening the Support System

To overcome insufficient resources, many participants spoke of the need to integrate outbreak responses. This should be initiated not only by those who are involved in VBD activities, but also from a position within the government. It is political will, some participants explained, that will lead to better actions from the people that follow them.

*“What is required is a political will and… a strengthening of technical and operational capacity at the local, departmental and national level.”*

—PD1

*“We would start from the central level, where there is a political union and there are suitable staff, with an interaction between country and territorial entities.”*

—I2

### 3.6. Information Consolidation

Two participants focused on the lack of consolidation of information within Colombia, and one went on to mention the importance of merging information not only from the government-based organisations but also other sectors, organisations, and institutions. Should a database of information be produced, it would provide empirical evidence to support the adaptation and implementation of public policies. It could also help to expedite the response to outbreaks: if information was present online it would allow for the analysis of a situation in real-time and thus enable appropriate responses to be made. This database could have all the data collected over the years by the health secretaries with information such as distribution of vector species in each department, number of cases reported, vector control activities conducted in the area, and information about insecticide resistance.

*“Firstly, they need to have the information online. So, it could be on the internet at the right moment. Like the system we have for the vital registrations (births and deaths), so the analysis could be done in present time. Instead, at the moment we need to wait for the consolidation data and the analysis is behind.”*

—I11

### 3.7. Further Research and Education

The panel discussions and interviews underlined the need to conduct more research and to make improvements in the academic sector. One participant mentioned the need to enhance and broaden the teaching for medical students as well as practicing clinicians, to ensure that VBDs are not solely understood from a clinical aspect, but from the entomological aspect as well.

*“To propose a single, very difficult strategy, is to start improving from the academy, because it touches… those professionals who are only focused on patient care… Many times the medical doctors don’t think that they have to know about tropical diseases and mosquitoes.”*

—PD1

Finally, as a future outlook on VBDs from a commercial point of view, a participant recommended more research into new chemical forms of control. This will help to replace old compounds that are currently being used and mitigate problems of resistance.

*“I think through PQT-VC (WHO Prequalification Team: Vector Control Products) all companies should join to improve new formulations, new strategies, and also to cooperate with the governments, because this is technology transfer.”*

—I6

## 4. Discussion

Our study began with an exploration of the roles of different stakeholders employed in vector control. It was necessary for our study to gain insights into the activities of these individuals, extending from those who carry out vector control on behalf of the health secretariats, to those from the academic and research sectors who make contributions to the improvement of vector control activities and contribute to community participation.

### 4.1. Delayed Responses to Vector Outbreaks

Control procedures, such as the use of insecticides, were largely seen by our stakeholders as being both delayed and reactive, with insufficient forward planning around expected outbreaks. These limitations in the epidemiological surveillance and control of VBDs combine with inadequate notification rates in some regions [8]. The incidence of VBD infections may be expected to follow seasonal patterns that would allow for predictions to be made and preparation to be set ahead of peak transmission periods, and some stakeholders indicated that such seasonal occurrence of VBDs was understood. However, we note that studies of the occurrence of dengue fever in Colombia have shown that cases do not always follow clear seasonal patterns [39], and the occurrence of major outbreaks is affected by such factors as the El Niño–Southern Oscillation [8]. Whilst temperature and rainfall do influence mosquito populations and vector-pathogen interactions [40,41,42,43], many other factors contribute to the development of outbreaks. Advances in predictive modelling using surveillance, with meteorological and socio-economic data will be required to improve forward planning, as promoted by some of the stakeholders interviewed [44].

### 4.2. Lack of Resources and Administrative Discontinuity

The responses from participants from Meta and Vichada indicate that there is strong use of the national protocols for vector control throughout these departments, but they are being applied to different environments, and the nuances of these differences are not reflected in the protocols. Further, some respondents indicated that the implementation of the protocols may be inadequate, may be interrupted through administrative changes, or simply hampered by a limitation of resources. The lack of prioritisation and investment in vector control is a common complaint that dates back decades, when successful *Aedes*-control campaigns of the past were replaced by reactive control during epidemics, which has been largely ineffective [45]. Given these challenges, it is important that Colombia has infrastructures in place to prevent the growth of vector populations that might give rise to outbreaks. For example, reliable water supplies in rural areas would reduce the need for people to have storage tanks on their properties that can be used as habitats for immature larvae, and where water has to be stored, suitable vector control should be implemented. A cluster randomized trial in Girardot found that long-lasting insecticide-treated water container covers, coupled with treated window and door curtains, reduced dengue vector density after a short-term evaluation [46]. Establishing sustained vector control through such tools could reduce the baseline vector population, and, even with conservative assumptions for efficacy, could be highly cost-effective [47]. The alternative approach of responding to outbreaks can be much more costly [48] and can be inhibited by the procedural delays our study respondents identified.

### 4.3. Communication between Organisations

Opportunities to enhance the speed at which responses can be mounted to VBDs, and the scale-up of vector control interventions, are expected to lead to proportionate reductions in disease cases [49] and may come from online technologies. For example, a prototype app-based platform has been developed by a team of information-communication technology experts from Ibagué, the capital city of Tolima in central Colombia [50]. The platform allows community users to report possible larval sites in the city, and for public health inspectors to provide georeferences for visits to such sites in real time. A second system, named VECTOS, which has been trialled in the municipalities of Giron, Yopal, and Buga, enables real-time monitoring of weekly epidemiological indicators, entomological indices, and social surveys [51]. Given the concerns with the delayed responses and integration between different organisations, we believe that closer collaboration and the adoption of such systems may enhance the response to future outbreaks.

### 4.4. Community Participation

The Colombian government, in collaboration with international organizations and NGOs, conducts public health campaigns to raise awareness about vector-borne diseases and to promote preventive measures. In some cases, local communities trust and participate in these campaigns, engaging in activities such as removing sites that could be used for larval development, distributing insecticide-treated bed nets, and educating their peers about disease prevention, which can lead to positive changes in perception and behavior regarding disease prevention. Unfortunately, the majority of residents do not participate, and lack care, or are apathetic about the matter [52].

An important element of preparedness is education and community awareness [53]. The response from several interview participants, that their most valuable contribution to vector control is education of current students, underlines a recognition that VBDs will remain an important issue in Colombia, and that a skilled workforce is crucial to future control efforts. Several university courses are available in Colombia that cover medical entomology and epidemiology, such as a tropical medicine course at Universidad Cooperativa de Colombia [54], which is designed to actively involve students within communities, and the Master of Science in entomology course at the Universidad Nacional de Colombia [55].

Our findings match those reported from other resource-limited countries experiencing VBDs. In Brazil, stakeholders perceived that prevention and control efforts performed by authorities during dengue outbreaks remained inadequate and required an upscaling of responses and enhanced coordination by governmental authorities [56]. In the Dominican Republic, members of public health and entomology teams perceived limitations similar to those reported in Colombia, including a shortage of resources, and limited community cohesion [57]. Case studies reviewed by Horstick et al. [58], from Brazil, Guatemala, The Philippines, and Vietnam similarly reported staffing levels, capacity building, management and organisation, funding, and community engagement as being insufficient, and a comparison of mosquito control programmes in seven urban sites in Kenya, Egypt, Israel, Costa Rica, and Trinidad also identified issues of a lack of inter-sector collaboration and sustainable funding for mosquito control [59]. The convergence of our findings with those of other studies likely relate to similar resource limitations in each country and the seemingly continuous threat of VBDs. By contrast, mosquito control districts in Florida report fruitful collaborations with research facilities, rapid interruption of Zika virus transmission in 2016, and a significant capability to handle potential mosquito-borne disease threats [60,61,62]. However, even Florida, one of the few states with extensive organized mosquito control programmes, experiences a perception of understaffing: slightly more than half of respondents in Florida mosquito control districts felt they were understaffed, which may compromise mosquito surveillance and control efforts in some districts [63].

It has been concluded elsewhere that countries worldwide face the same challenges relating to human, financial, and structural resources in the surveillance and control of VBDs [64], so the limitations described here for Colombia need to be understood in the context of a global problem that demands attention to strengthen control programmes and enhance VBD research.

Through analysis of the stakeholder consultations, we recommend a consolidation of information and improved data sharing, as well as better communication between the various bodies involved in vector surveillance and control in Colombia. A more adaptable system would allow for local authorities to implement the most suitable control methods in their municipalities. We also agree with the participants that greater engagement with local communities, and continued funding for education and research activities, are required. We note that these recommendations are broadly in alignment with those of integrated vector management approaches (IVM), which have the following five key elements: (i) evidence-based decision-making, (ii) integrated approaches, (iii), collaboration within the health sector and with other sectors, (iv) advocacy, social mobilization, and legislation, and (v) capacity-building. This approach has been strongly advocated for the control of malaria in Africa [65,66].

There is a lack of qualitative studies in the Americas that explore the perspectives of those involved in vector control and VBD research. Future studies should attempt to gain the perspectives of at least one person from each department, as well as other professional profiles such as policy makers or health workers, which would help to broaden the findings of the present investigation and reach data saturation. In particular, it would be valuable to capture the opinions of indigenous populations who may have different experiences and outlooks and may have different expectations from government authorities.

### 4.5. Limitations

We recognise that interviewers may have influenced participant answers in some way, considering that they each had a personal affiliation with one or more of the authors. The content analysis approach we used allowed for flexibility because it did not rely on a codebook or categories defined prior to the analysis. Deductive content analyses may miss important content that does not fit into such predetermined categories and might overlook new insights or ideas that emerge from the data. Instead, emerging categories that may not have been considered at the outset were created as we became familiar with the content of the transcribed text. This approach is also not without limitations and may be influenced by researcher’s personal biases and preconceptions, and by their experience. To limit inaccuracies and to ensure that no relevant data had been excluded, credibility checks were made by different co-investigators to those conducting the primary analysis [36,67].

With regards to transferability, we note that the stakeholders interviewed and providing discussion in the current study were from Bogotá and just three out of the 32 departments of Colombia. We acknowledge that a larger sample size and broader sampling from across the country may have provided a more diverse range of opinions and could potentially have generated more refined and detailed results. In particular, it would be valuable to interview stakeholders from other parts of the Central East region, which has historically reported the most cases of dengue [8], and Amazonia and the Pacific coast, which experience malaria epidemics [68]. Is important to complement this information with anonymous online surveys that could help us better understand the vector control situation in this part of the country.

## 5. Conclusions

This study illustrates the perceptions and experiences of stakeholders towards vector control and vector-borne diseases in a relatively wealthy region of Colombia. Although our consultations were limited in number, we found valuable insights and alignment across both the panel discussions and the interviews, with the majority in agreement that there is a concern over the disconnection between governing bodies, and a shortage of resources. The sessions demonstrated the need for more collaborative actions, a more balanced distribution of resources, and more engagement with communities. Because our findings are supported by those reported elsewhere in the literature, we believe that many of our stakeholder recommendations are transferrable to other settings.

Our study could be a stimulus for the authorities to adapt their protocols to make them more suitable for different communities. Arboviruses are an expression of social inequalities and inequities, so the problem must be addressed from the social determinants. Actions should be focused on strengthening programs to reduce vector density and transmission of vector-borne diseases, as well as increasing diagnostic capacity and clinical and epidemiological surveillance, strengthening research, and improving education, while taking into account the cultural and social differences and respecting the communities. Most important is the political will to change the way the limited resources are managed.

## Figures and Tables

**Table 1:** Characteristics of participants involved in the semi-structured interviews.

Interview Number	Place of Employment	Location
I1	Public health surveillance group of the Departmental Secretary of Health (DSH)	Meta
I2	Departmental Secretary of Health	Guaviare
I3	Departmental Secretary of Health—Entomology Laboratory	Guaviare
I4	Departmental Secretary of Health—Entomology Laboratory	Meta
I5	Departmental Secretary of Health—Entomology Laboratory	Meta
I6	Vector & pest management private company	Bogotá
I7	Departmental Secretary of Health—Entomology Laboratory	Vichada
I8	Ministry of Health and Social Protection	Bogotá
I9	Expert advisor	Bogotá
I10	Universidad el Bosque	Bogotá
I11	National Institute of Health	Bogotá
I12	National Institute of Health	Bogotá

**Table 2:** Characteristics of participants involved in the panel discussions.

**Panel discussion 1 (PD1):** The governmental entity, the private sector and higher education against arboviruses and their control		
**Profession**	**Place of employment**	**Country**
Manager	Vectors & Pest Management	Colombia
Entomologist PhD	London School of Hygiene & Tropical Medicine	United Kingdom
Entomologist specialist	Departmental Secretary of Health of Meta—entomology laboratory	Colombia
MD. Medicine faculty dean	Universidad Cooperativa de Colombia	Colombia
Biologist/entomologist PhD	Moderator	Colombia
**Panel discussion 3** (PD3): Role of the society in the control and prevention of arboviruses transmitted by Aedes		
Anthropologist	Departmental Secretary of Health of Meta	Colombia
Anthropologist	Departmental Secretary of Health of Meta	Colombia
Environmental engineer	Departmental Secretary of Health of Guaviare—Entomology laboratory	Colombia
MD—public health PhD	Moderator	Colombia

**Table 3:** Categories and subcategories of analysis.

Category	Definition	Subcategories (Pre-Defined and Emerging)
Involvement in vector control		Process of vector control actions Types of tools Effectiveness of surveillance system
		Context (political) Management and Financing Data sharing Data source integration Implementation of interventions Type of outbreak response Sustainability of actions
		Institutions involved in vector control Joint work between institutions and community
Limitation of vector control		Innovative interventions Vector biology Vector behaviour Integration of information
Participant recommendations of vector control	Recommendations developed based on expert opinion	

## Data Availability

The datasets used and/or analysed during the current study are available from the corresponding author on reasonable request. Informed consent was obtained from all subjects involved in the study.

## Supplementary Materials

The following supporting information can be downloaded at: https://www.mdpi.com/article/10.3390/tropicalmed9020043/s1, Additional File S1: Question guide for interview session; Additional File S2: first regional and international meeting of experts in control of arboviruses transmitted by *Aedes aegypti*; Additional File S3: Pannel Discussion 1 and 3.
