# Supplementary material for: A Qualitative Analysis of the Perceptions of Stakeholders Involved in Vector Control and Vector-Borne Disease Research and Surveillance in Orinoquia, Colombia
Source: Trop Med Infect Dis. 2024 Feb 6;9(2):43. doi: 10.3390/tropicalmed9020043 (PMC10892243; doi:10.3390/tropicalmed9020043)
Supplement: Supplementary file 1 [file tropicalmed-09-00043-s001.zip › tropicalmed-2721510-supplementary.pdf]

Additional file S1

Question guide for interview session.

Introduction

1. Please could you briefly state your job title
2. Are you directly involved in the management of and/or development of vector control policies within Colombia? – If so, please provide details on what your role entails.
3. If you are more involved in research, please outline your main areas of interest and any key findings you have made.

Vector control and surveillance activities

4. What are the main tools of vector control currently used in your district, and elsewhere in Colombia?
5. Is there a surveillance system currently in place for vectors of disease? If so, what is the current surveillance system?
6. Is there a surveillance system currently in place for VBDs? If so, what is the current surveillance system?
7. Do you know of activities to assess the value of vector-control tools? (e.g. insecticide resistance assessments to determine which bed nets are most suitable)
8. How efficient is the process of detecting a VBD outbreak?
9. What would you deem to be the main limitations in Colombia in regards to vector control and the ability to respond to outbreaks?
10. Do you have any data that you would like to share? (e.g. insecticide resistance data, mosquito trapping data)

Politics, funding

11. Who are the fundamental collaborating organisations involved in vector control in Colombia?
12. How do you think the current vector control implementation, data sharing and management could be improved?

Limitations and future research

13. Given your expertise and your experience, what do you believe are key knowledge gaps in the implementation of efficient and effective vector control (e.g. which tools are effective, which tools are well received by communities)?
14. Focusing in on the 'vector' aspect, do you believe there are gaps in knowledge within the research community on vector biology and behaviour (e.g. response to particular insecticides)?
15. What recommendations would you make to help improve vector control and bridge knowledge gaps in Colombia?
16. What are your main concerns about VBDs in the future (e.g. climate change, urbanization, migration from neighbouring countries, resistance to control tools, political instability)?
17. What do you see in the future for vector control in Colombia (e.g. novel tools, genetic modification, improved infrastructure)?

Additional file S2: first regional and international meeting of experts in control of arboviruses transmitted by Aedes aegypti

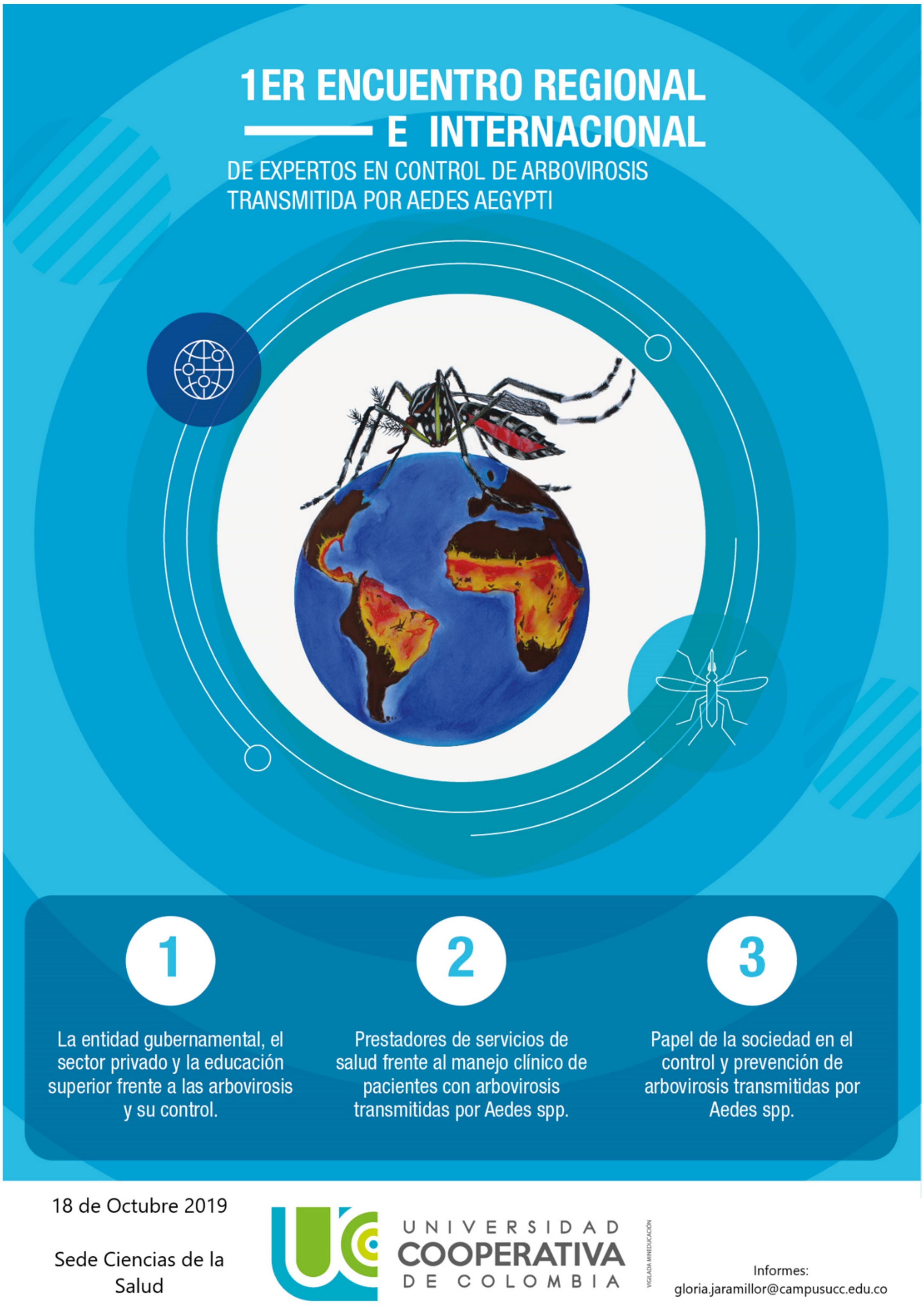

Additional file S3: Pannel Discussion 1 and 3.

Panel Discussion 1: THE GOVERNMENT ENTITY, THE PRIVATE SECTOR AND HIGHER EDUCATION AGAINST ARBOVIRUSES AND THEIR CONTROL

From their experience and field of action, which is the most important contribution made to the control of arbovirus

What has been the greatest difficulty he has had to face in the control of these diseases and their vectors and how he overcame it.

Do you think vector control programs and their arboviruses have failed? And Who or who you believe have been most responsible

What do you think is the best strategy to reduce the incidence of these arboviruses and improve control programs.

How it would integrate public, private and academia into a viable strategy of vector control and arbovirus decline.

Panel Discussion 3: ROLE OF SOCIETY IN THE CONTROL AND PREVENTION OF AEDES-TRANSMITTED ARBOVIRUS

In your professional experience, how important is the linking of communities in vector control?

What strategies have you observed as most effective in community work for the control and prevention of arbovirus?

Faced with intersectoral actions: Which sectors are key to improving the effectiveness of interventions?

How to manage the cultural imaginary that prioritizes chemical control over other actions?

How to deal with control and prevention in indigenous communities?

Within the life cycle approach, who should be prioritized in education and prevention activities? (School population, Adolescents and young people, heads of household, older adults)

The Colombian health system is facing a structural crisis, within which are administrative or economic barriers to access to services (Authorizations, co-payments, moderating fees, opportunity in care). How to handle this issue in social management?
